# Necrobiotic Xanthogranuloma Associated With IgG Kappa Smoldering Myeloma Presenting as Vision-Threatening Orbital Disease: Clinical Response to Plasma Cell-Directed Therapy

**DOI:** 10.7759/cureus.111170

**Published:** 2026-06-19

**Authors:** Hira I Cheema, Maurizio Zangari, Carolina Schinke, Frits Van Rhee, Sharmilan Thanendrarajan

**Affiliations:** 1 Hematology and Oncology, University of Arkansas Medical Center, Little Rock, USA; 2 Hematology and Oncology, University of Arkansas for Medical Sciences, Little Rock, USA

**Keywords:** necrobiotic xanthogranuloma, non-langerhans histiocytic disorder, orbital disease, plasma cell therapy, smoldering myeloma

## Abstract

Necrobiotic xanthogranuloma (NXG) is a rare chronic non-Langerhans cell histiocytosis characterized by xanthomatous granulomatous lesions and a strong association with monoclonal gammopathies, particularly IgG-kappa paraproteinemia. Periorbital involvement is common and may lead to orbital mass effect, visual compromise, and significant morbidity. We report the case of a 68-year-old woman who presented with progressive visual decline, choroidal folding, orbital compression, and ulcerative periorbital lesions. Biopsies of the right upper eyelid and lower extremity confirmed NXG. Evaluation demonstrated an IgG-kappa monoclonal gammopathy with bone marrow findings consistent with smoldering multiple myeloma. Given vision-threatening orbital disease, plasma cell-directed therapy was initiated despite the absence of myeloma-defining events. The patient received sequential treatment with daratumumab-bortezomib-dexamethasone, cyclophosphamide-lenalidomide-dexamethasone, cyclophosphamide-dexamethasone, and later isatuximab-carfilzomib-dexamethasone. Although the best hematologic response achieved was partial remission, she experienced near-complete resolution of cutaneous and periorbital lesions with stabilization of ocular symptoms. This case supports the concept of NXG as a monoclonal gammopathy of clinical significance and highlights that plasma cell-directed therapy may produce substantial clinical benefit even without deep hematologic remission.

## Introduction

Necrobiotic xanthogranuloma (NXG) is a rare chronic granulomatous disorder classified within the spectrum of non-Langerhans cell histiocytoses [1,2]. It most commonly presents as yellow-orange papules, plaques, or nodules that may ulcerate, scar, and progressively enlarge over time [1,2]. The periorbital region is frequently affected, although lesions may also involve the trunk, proximal extremities, and extracutaneous organs [1-4].

Histopathologically, NXG is characterized by palisading granulomatous inflammation surrounding areas of necrobiotic collagen, accompanied by foamy histiocytes, cholesterol clefts, lymphoplasmacytic infiltrates, and multinucleated giant cells, including Touton-type giant cells [1,2]. Recently proposed diagnostic criteria incorporate characteristic cutaneous lesions, compatible histopathology, and supporting features such as paraproteinemia, plasma cell dyscrasia, lymphoproliferative disorder, or periorbital distribution [2]. A strong association exists between NXG and monoclonal gammopathies. Large series and systematic reviews have demonstrated frequent paraproteinemia, with IgG-kappa among the most commonly reported monoclonal protein subtypes [1-4]. Associations with monoclonal gammopathy of undetermined significance, smoldering multiple myeloma, multiple myeloma, and other lymphoproliferative disorders have been described [2-4].

Ocular involvement may include periorbital plaques, orbital masses, proptosis, restricted ocular motility, eyelid malposition, ocular surface disease, inflammatory eye disease, and visual loss from inflammatory or compressive mechanisms [2-4]. The pathogenesis remains incompletely understood, but studies suggest that monoclonal immunoglobulins may interact with lipoproteins and promote macrophage activation, lipid accumulation, and granulomatous inflammation [5-7]. This biologic link supports viewing NXG within the framework of monoclonal gammopathy of clinical significance, in which a small plasma cell or B-cell clone causes clinically significant organ injury despite not meeting criteria for overt malignancy [5].

Because NXG is rare, there are no established consensus treatment guidelines. Reported therapies include corticosteroids, alkylating agents, immunomodulatory drugs, intravenous immunoglobulin, radiation therapy, surgery, and plasma cell-directed therapy, with variable responses [8-12]. We report the case of a patient with vision-threatening orbital NXG associated with IgG-kappa smoldering multiple myeloma who achieved marked clinical improvement following sequential plasma cell-directed therapy.

## Case presentation

**Figure 1:**
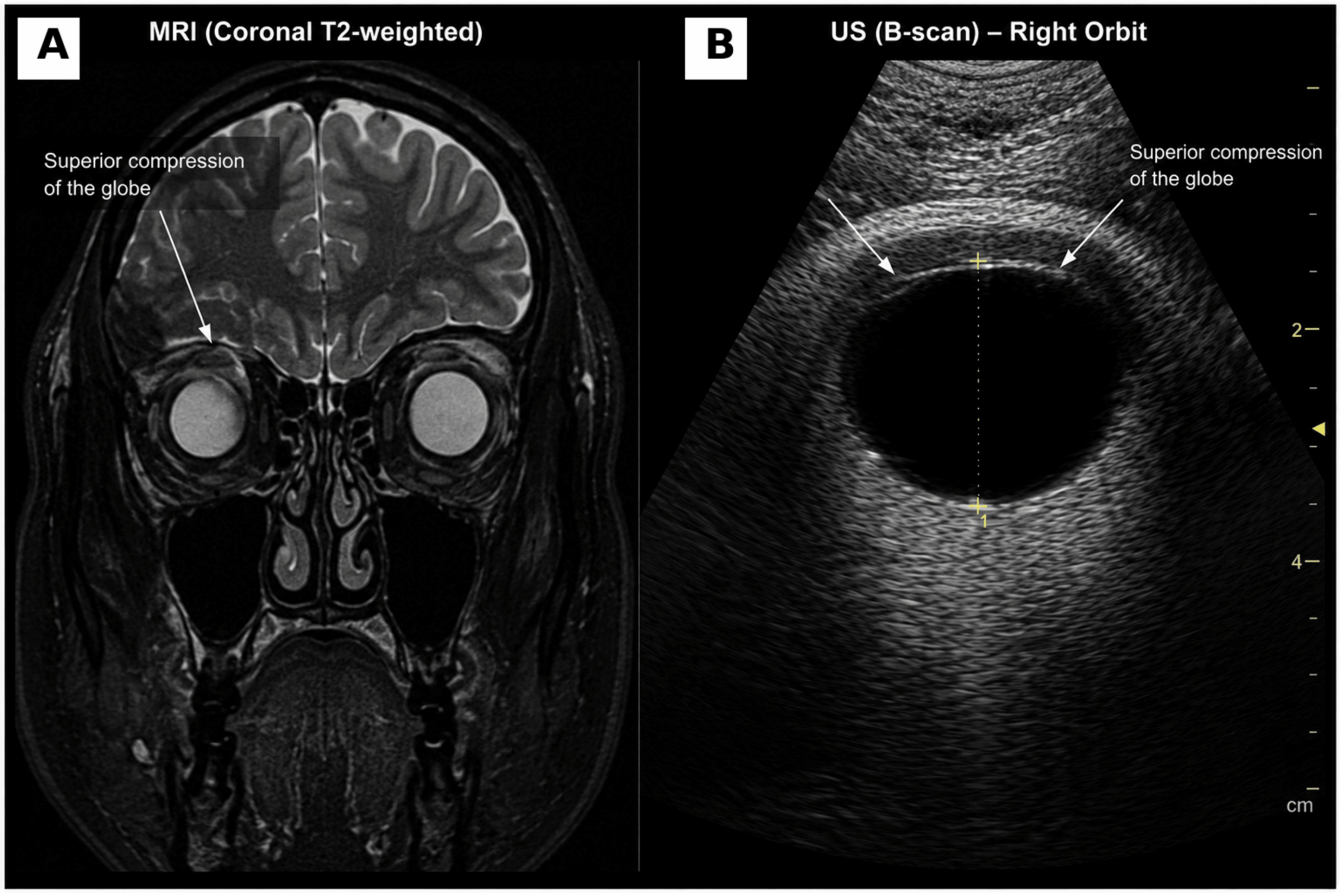
Orbital imaging demonstrating superior compression of the right globe (A) Coronal T2-weighted MRI showing superior orbital soft tissue causing downward mass effect on the globe. (B) B-scan ultrasonography demonstrating flattening of the superior scleral contour consistent with external compression by orbital disease MRI: magnetic resonance imaging

The patient was a 68-year-old woman with a past medical history of chronic ocular disease, including cataracts, no history of autoimmune disorders or recreational drug abuse or addictions. Her family history was unremarkable. She was found to have choroidal folding of the right eye in July 2024. Although her vision initially improved, she subsequently developed progressive blurred vision, ocular pain, and floaters involving the right eye. Ophthalmologic examination revealed an ulcerative lesion of the right upper eyelid with increased resistance to retropulsion. Orbital ultrasonography demonstrated superior compression of the globe. MRI confirmed an orbital mass producing downward mass effect on the superior aspect of the right globe (Figure 1).

**Figure 2:**
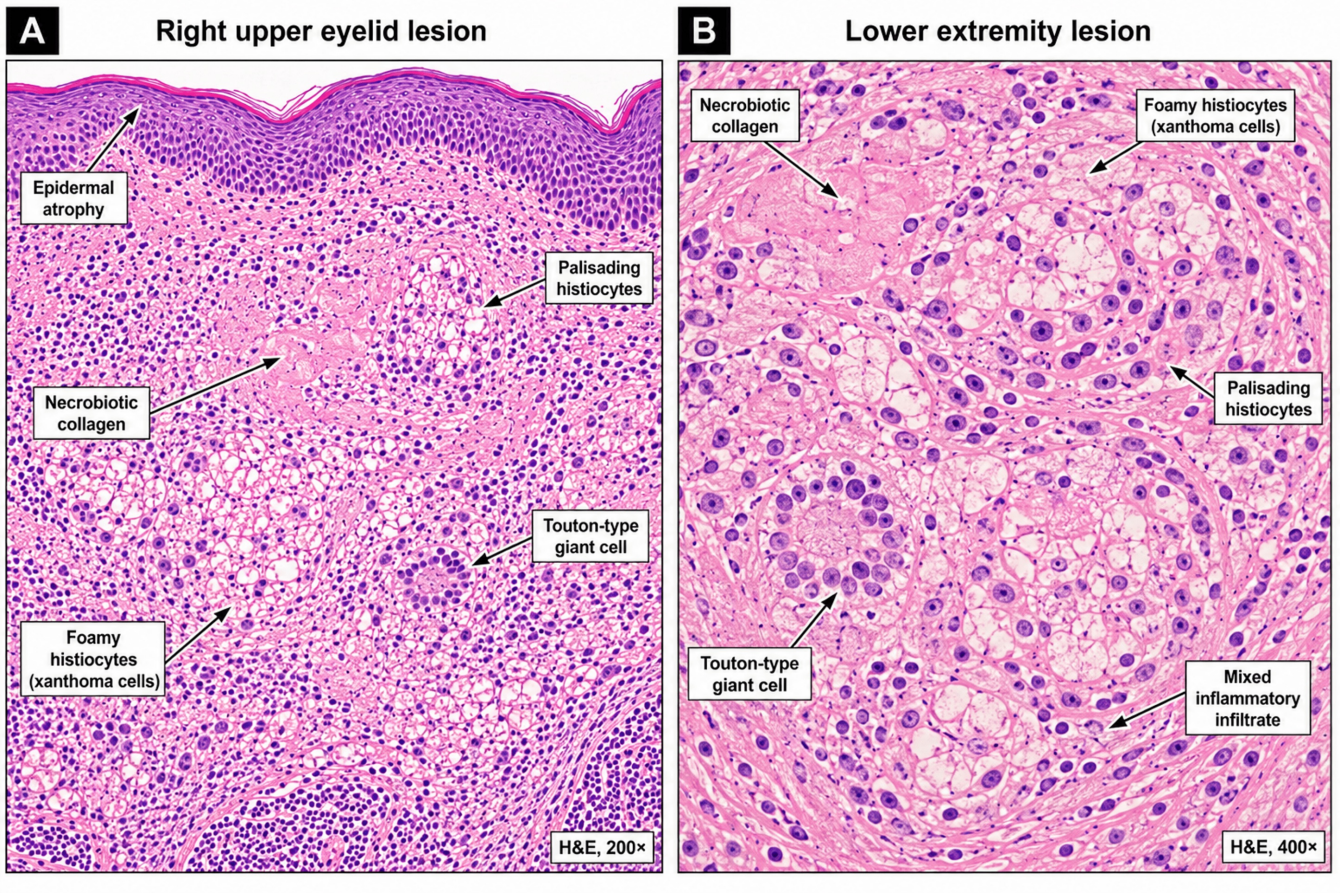
Histopathologic findings of necrobiotic xanthogranuloma (A) Right upper eyelid biopsy showing palisading granulomatous inflammation with foamy histiocytes, necrobiosis, and Touton-type giant cells. (B) Lower extremity biopsy demonstrating extensive necrobiosis, cholesterol clefts, multinucleated giant cells, and xanthogranulomatous inflammation

Multiple placoid cutaneous lesions were also identified on the extremities and trunk. Biopsies obtained from the right upper eyelid and a lower extremity lesion demonstrated findings consistent with NXG, including palisading granulomatous inflammation, necrobiosis, foamy histiocytes, cholesterol clefts, and Touton-type giant cells (Figure 2).

Laboratory evaluation revealed an IgG-kappa monoclonal protein with serum IgG of 3,160 mg/dL. Bone marrow biopsy initially demonstrated approximately 5% atypical plasma cells. Whole-body imaging demonstrated no lytic lesions or other myeloma-defining abnormalities. She was initially treated with prednisone 60 mg daily, followed by a gradual taper. Further hematologic evaluation in October 2024 demonstrated an M-protein of 1.9 g/dL with positive serum immunofixation. Urine immunofixation revealed minimal Bence Jones proteinuria with free kappa light chains. Serum kappa free light chain level was 11 mg/dL with a kappa/lambda ratio of 14.

**Figure 3:**
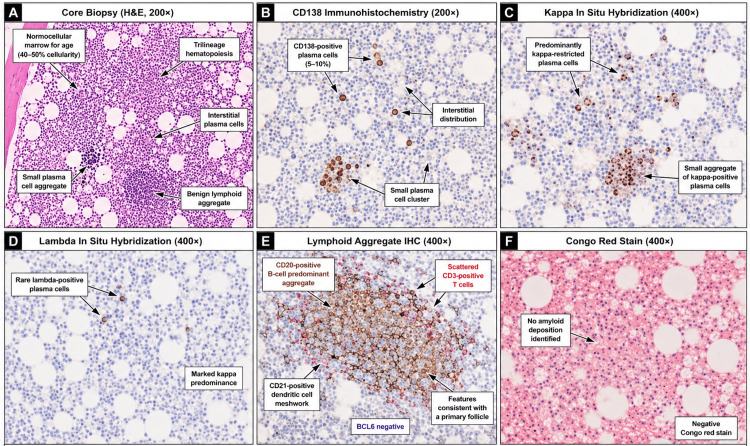
Bone marrow pathology demonstrating IgG-kappa plasma cell neoplasm (A) H&E-stained core biopsy. (B) CD138 immunohistochemistry. (C) Kappa in situ hybridization. (D) Lambda in situ hybridization. (E) Lymphoid aggregate immunophenotyping. (F) Congo red stain negative for amyloid deposition

Repeat bone marrow examination demonstrated normocellular marrow for age, with 40-50% cellularity and preserved trilineage hematopoiesis. Plasma cells comprised approximately 7% of marrow aspirate cellularity and 5-10% of marrow cellularity on core biopsy. CD138 immunohistochemistry highlighted plasma cells distributed primarily as individual interstitial cells and small aggregates. In situ hybridization demonstrated kappa light-chain predominance. Most plasma cells expressed IgG, whereas IgG4-positive plasma cells were exceedingly rare. Congo red staining was negative for amyloid deposition (Figure 3).

Imaging remained negative for myeloma-defining bone lesions. Chronic thoracic compression deformities involving T1, T3, T4, and T5 were considered longstanding and unrelated to active myeloma. Based on these findings, the patient was diagnosed with IgG-kappa smoldering multiple myeloma. Because the patient had progressive, vision-threatening orbital NXG associated with a plasma cell dyscrasia, treatment was directed toward the underlying plasma cell clone despite the absence of myeloma-defining events, consistent with the monoclonal gammopathy of clinical significance framework [5].

On January 7, 2025, treatment with daratumumab, bortezomib, and dexamethasone was initiated. Lenalidomide was initially avoided because of thrombotic risk following a recent hip fracture. Due to only a minor response, therapy was discontinued on March 18, 2025, and replaced with cyclophosphamide, lenalidomide, and dexamethasone. Treatment was interrupted multiple times because of recurrent infections. Following a cerebrovascular accident, lenalidomide was discontinued on July 8, 2025, and cyclophosphamide-dexamethasone was continued. The patient subsequently received isatuximab, carfilzomib, and dexamethasone, which was discontinued in January 2026.

**Figure 4:**
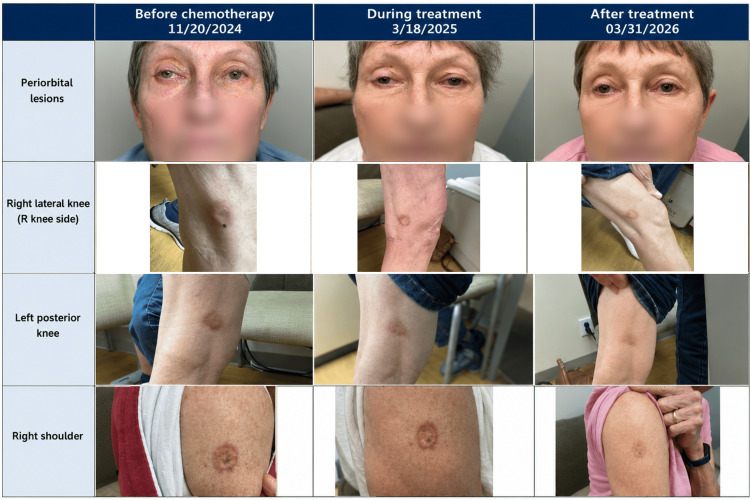
Clinical evolution of necrobiotic xanthogranuloma lesions Clinical evolution of necrobiotic xanthogranuloma lesions involving the periorbital region, right lateral knee, left posterior knee, and right shoulder before chemotherapy (November 20, 2024), during treatment (March 18, 2025), and after treatment (March 31, 2026), demonstrating marked regression of cutaneous and periorbital disease following plasma cell-directed therapy

Despite the cessation of treatment, the patient remained clinically stable during the following 2.5 months. Laboratory evaluation demonstrated M-protein 1.3 g/dL, kappa free light chain 7.27 mg/dL, and kappa/lambda ratio 10.85. The best hematologic response achieved was partial remission. Remarkably, cutaneous and periorbital NXG lesions demonstrated near-complete resolution, accompanied by stabilization of visual symptoms and substantial improvement in clinical status (Figure 4). At follow-up, she was noted to be in the best overall clinical condition observed during several years of treatment.

## Discussion

NXG is an exceptionally rare non-Langerhans cell histiocytosis. The largest contemporary study by Nelson et al. combined a multicenter cohort with a systematic review and identified 235 patients worldwide, underscoring the rarity of this disorder [1]. Likewise, Hilal et al. reported only 35 patients over 30 years at a tertiary referral center, with paraproteinemia identified in most patients and monoclonal gammopathy representing the most common associated hematologic abnormality [2]. Orbital involvement has been described in smaller case series and isolated reports and may manifest as periorbital plaques, orbital masses, proptosis, restrictive ophthalmoplegia, or visual loss [7,8].

Presentation with superior globe compression accompanied by choroidal folding, as seen in our patient, is distinctly uncommon and has rarely been emphasized in prior reports. Furthermore, there is limited published experience describing sequential modern plasma cell-directed therapy, including anti-CD38 monoclonal antibodies, in patients with NXG associated with smoldering myeloma. The marked clinical response observed in our patient despite only partial hematologic remission further highlights the unique features of this case.

This case highlights several important aspects of NXG. First, the patient fulfilled proposed diagnostic criteria through the presence of characteristic cutaneous lesions, compatible histopathology, periorbital involvement, paraproteinemia, and an associated plasma cell dyscrasia [2]. Second, the case demonstrates the well-recognized association between NXG and monoclonal gammopathies. Large retrospective series and systematic reviews have consistently shown frequent paraproteinemia, with IgG-kappa among the most common monoclonal protein subtypes [1-4]. Although the precise mechanism remains uncertain, monoclonal immunoglobulins are thought to contribute to macrophage activation, abnormal lipid handling, and granulomatous inflammation, providing a biologic rationale for targeting the underlying plasma cell clone [6,7].

The histopathologic differential diagnosis of NXG includes several granulomatous disorders, most notably necrobiosis lipoidica, granuloma annulare, juvenile xanthogranuloma, Erdheim-Chester disease, rheumatoid nodules, and infectious granulomatous disorders [1,4]. Necrobiosis lipoidica may demonstrate palisading granulomas and collagen degeneration but typically lacks the extensive cholesterol clefts and prominent Touton giant cells characteristic of NXG. Granuloma annulare is distinguished by abundant dermal mucin deposition and the absence of xanthomatous inflammation. Juvenile xanthogranuloma may contain Touton giant cells but generally lacks necrobiosis and paraproteinemia. Erdheim-Chester disease is another non-Langerhans cell histiocytosis that may demonstrate foamy histiocytes and giant cells; however, it is usually associated with characteristic systemic manifestations, including long-bone sclerosis and frequent BRAF mutations [5]. In our patient, the presence of extensive necrobiosis, cholesterol clefts, Touton-type giant cells, periorbital involvement, and an associated IgG-kappa monoclonal gammopathy strongly supported the diagnosis of NXG.

The patient met the criteria for smoldering multiple myeloma rather than active myeloma because she lacked myeloma-defining CRAB features or myeloma-defining biomarkers. Nevertheless, her plasma cell disorder was associated with significant organ damage through progressive orbital NXG. This presentation is consistent with monoclonal gammopathy of clinical significance, where treatment is justified by clinically significant organ injury rather than tumor burden alone [5]. Orbital involvement represents one of the most serious manifestations of NXG. Previous studies have reported orbital masses, proptosis, ocular motility restriction, eyelid malposition, ocular surface morbidity, and visual loss [2-4]. Our patient demonstrated orbital mass effect with superior globe compression and choroidal folding, highlighting the potential for vision-threatening complications if treatment is delayed.

Management of NXG remains challenging because prospective clinical trials are lacking. A systematic review demonstrated substantial heterogeneity in treatment approaches, including corticosteroids, alkylating agents, immunomodulatory drugs, intravenous immunoglobulin, radiation therapy, and surgery [8]. Surgical excision is generally discouraged except in selected vision-threatening circumstances because recurrence is common [2,8]. Several reports have demonstrated the efficacy of plasma cell-directed or immune-modulating therapy in NXG associated with paraproteinemia. Responses have been reported with thalidomide-dexamethasone and lenalidomide-dexamethasone [9,10]. Intravenous immunoglobulin has also shown encouraging activity in selected patients with periorbital disease [11,12].

Perhaps the most notable aspect of this case is the discordance between hematologic and clinical responses. Although only partial hematologic remission was achieved, the patient experienced near-complete resolution of cutaneous lesions and marked improvement in orbital disease. This observation suggests that suppression of the pathogenic clone may be sufficient to control NXG-associated inflammatory tissue injury even without deep hematologic remission. The dramatic clinical response observed in this case supports the concept that NXG should be considered a manifestation of monoclonal gammopathy of clinical significance when associated with paraproteinemia. Recognition of this relationship may justify earlier institution of systemic therapy, particularly in patients with vision-threatening disease.

## Conclusions

NXG is a rare but potentially vision-threatening manifestation of monoclonal gammopathy. This case report demonstrates that plasma cell-directed therapy can produce substantial clinical benefit despite only partial hematologic response. Recognition of NXG as a manifestation of monoclonal gammopathy of clinical significance is important because treatment of the underlying plasma cell clone may prevent irreversible organ damage even when criteria for symptomatic multiple myeloma are not met.
